# Exploring Sociodemographic Correlates of Fathers’ and Mothers’ Behavioral Control

**DOI:** 10.3390/bs14121203

**Published:** 2024-12-15

**Authors:** Xiaoyan Xu, Zahyah Hanafi, Nordin Abd Razak

**Affiliations:** 1 School of Education, Shandong Women’s University, Jinan 250300, China; 2 School of Education, Faculty of Social Sciences and Leisure Management, Taylor’s University Lakeside Campus, Subang Jaya 47500, Malaysia; zahyah.hanafi@taylors.edu.my (Z.H.); nordin.abdrazak@taylors.edu.my (N.A.R.)

**Keywords:** behavioral control, gender, educational level, income

## Abstract

Parental behavioral control is one of the most common parenting practices employed by parents in China. It is necessary to identify this practice and how it may be influenced by adolescents’ and parents’ sociodemographic factors in order to develop family intervention strategies. The present study examined whether fathers’ and mothers’ behavioral control was related to adolescents’ gender, age, and sibling status and parents’ age, education level, and income. A sample of 916 adolescents, aged 11–15 years, reported their fathers’ and mothers’ parental behavioral control. In terms of adolescents’ gender, boys perceived higher level of fathers’ behavioral control compared to girls. Regarding parents’ sociodemographic factors, the level of mothers’ behavioral control was higher compared to that of fathers. Mothers’ education levels were reported to have a negative relationship with maternal behavioral control. Regarding adolescents’ gender differences, there was a positive relationship between fathers’ educational levels and behavioral control in adolescent boys. However, there was a positive relationship between fathers’ monthly income and fathers’ behavioral control in adolescent girls rather than boys. In addition, there was a negative relationship between mothers’ age and behavioral control in adolescent girls but not in boys. These findings add to the literature on Chinese mothers’ and fathers’ parenting and offer practical implications for Chinese family interventions.

## 1. Introduction

Parental behavioral control describes parents’ attempts to control their children through regulating, organizing, and supervising children’s performance and activities [1,2,3]. It is one of the key forms of parental control [2]. The current study sheds light on parental behavioral control in China. There are several reasons for this observation. First, compared to parents in Western countries, Chinese parents exhibit more behavioral control over their children, including influencing their decisions regarding what to wear and what kind of friends to make [4]. The differences in the level of parental behavioral control between Western and Chinese parents in child-rearing may be rooted in cultural differences between the West and East. Independence-oriented values are prevalent in Western culture. One tends to view oneself and others according to one’s own cognition and emotions [5]. Parents and children are independent of each other. Western parents merely value academic achievements. They are inclined to encourage their children to explore multiple fields and develop individuality [6]. Consequently, Western parents may have less discipline and control over their children. Chinese society reveres Confucianism and values inter-personal relatedness over autonomy [7]. Traditional Chinese culture places great value on honoring ancestors and families. The achievement and success of their offspring have become the primary goal of Chinese parents [8,9]. As such, when parents place their value on their children’s achievement, they may strive to control their children’s behaviors in order to make their children’s failures as small as possible [6,10]. Moreover, Confucianism emphasizes family hierarchy, highlighting the importance of obedience to parents and the role of parents in supervising and guiding children [8]. Frequent parental behavioral control is considered reasonable by Chinese fathers and mothers. However, the exploration of parental behavioral control in China is inadequate.

Second, the identification of the precursors of parental behavioral control has been neglected in China. Although many studies have focused on demographic and sociological variables related to parental behavioral control in Western families [11,12], the findings may not be applicable to China. Parental behavioral control is often conceptualized as parental monitoring in the West [12,13]. However, Chinese parents commonly use solicitation and restrictions to exert control over their children’s behavior [3]. There have been considerably fewer studies on parental behavioral control than on other parental practices in China. For example, there have been more investigations on parental psychological control than on parental behavioral control. What are the sociodemographic characteristics of parental behavioral control in China? Answers to this question are necessary and urgent, as they are a prerequisite to develop effective actions for parenting prevention and intervention. In view of these limitations, the present study aimed to examine whether the parental behavioral control of Chinese parents varies according to sociodemographic characteristics.

Belsky’s “process model” of parenting identifies that the personal resources of parents and the characteristics of children are closely related to parental function [14]. According to the family systems theory, the family is a complex entity with interdependent family members exerting an influence on each other [15]. As such, parental practices may vary by child and parental factors. Next, several potential child and parental factors that correlate with parental behavioral control are introduced.

Regarding adolescent factors, gender, age, and the number of siblings may be associated with parental behavioral control. Several studies have found that parents perform more behavioral control on Chinese boys than girls [16,17,18]. Since adolescent boys may be more impulsive [19], parents may consider providing proper guidance through behavioral control. In terms of adolescents’ age, adolescents’ perceived family functioning is lower in eighth grade than in seventh grade [20]. Another study found that the level of parental behavioral control decreases during early adolescence in Chinese society [17]. To the best of our knowledge, there is no direct evidence of a relationship between the number of siblings and parental behavioral control. With changes in China’s birth policy, an increasing number of children have sisters or brothers. The implications of sibling status are attracting increasing attention. Some studies have explored the linkage between sibling status and parenting [21]. However, due to limited existing research, it is necessary to examine whether parental behavioral control differs according to the number of siblings.

In terms of parental factors, studies have found that factors such as parents’ gender, age, education, and income may be relevant in how they raise their children. Differences between fathering and mothering have been reported in several studies. In general, mothers’ parenting practices are believed to be more favorable than those of fathers [22], while fathers are more authoritarian than mothers [23]. Findings have also revealed that mothers have a higher level of behavioral control compared to fathers in western countries [23,24]. A similar conclusion has been reached in China [17]. One reason for this phenomenon may be the natural obligation of mothers to take on responsibility for raising children. Moreover, in Chinese societies, fathers are considered to have a powerful and financially capable role. Fathers are not expected to take on a lot of parenting responsibilities but are usually the leaders in a traditional Chinese family [17]. In contemporary Chinese society, with the decline in the birth rate and the promotion of scientific parenting, children are gaining increasing priority. Both fathers and mothers today are willing to commit more time and energy to parenting compared to previous generations. China may witness a new shift in the variation in parenting behaviors between fathers and mothers [17]. This study aims to examine these differences further.

Research on the relationship between parental age and parental behavioral control is scarce. Theoretically, as parents age, they may gain more experience and become more responsible and have more emotional control [25]. One study noted that older fathers may have higher levels of parenting involvement [26]. Parental education level and income are closely related to parenting practices. In a study on urban African American mothers, the results demonstrated that mothers with lower levels of education tended to exhibit less sensitivity and more controlling behaviors [27]. Parental education level is positively related to parental control in Iranian society as well [28]. Parents with low education levels may not use parental monitoring effectively [13]. Similarly, some researchers have found that parental economic and social status is positively correlated with authoritative parenting but negatively correlated with authoritarian parenting in China [29]. The higher the level of the education of the father or mother is, the more behavioral control is exercised [30]. A study from Mainland China showed that for parents of middle school students, the lower the parental education level and income are, the more controlling behaviors the parents display [31]. Studies have shown that parents with low economic and social status raise their children with relatively higher levels of parental discipline [32]. When parents are less educated and financially stable, they might be more negative in parenting, such as being overcontrolling [33]. Patients with schizophrenia from poor families also report more neglect and less parental control [34]. Based on the family stress model, when a family is under economic stress, parents might experience emotional distress and engage in more negative parenting practices [35]. However, there is insufficient research on the direct influence of economic level on parental behavioral control [36]. Therefore, this study will focus on this aspect.

There may be adolescent gender differences in the relationship between parental factors and parental behavioral control. Parents may be more active in parental involvement and practices with children of their sex. Findings have shown that fathers exert stronger paternal behavioral control on adolescent boys than girls and mothers exert stronger maternal behavioral control on adolescent girls than boys [17]. Similarly, boys are more likely than girls to receive discipline from their fathers in Chinese families [37]. From a sociocultural perspective, Chinese fathers usually have high expectations of their sons [8]. In China, a father’s responsibility for the upbringing of a boy is primarily achieved by setting rules. That is, Chinese fathers may use more behavioral control over their sons than daughters. Gender socialization theories suggest that parents may be more involved in the socialization of children of their sex [38]. Accordingly, parental background factors may be related to rearing behaviors on children of the same sex. For instance, Brazilian girls aged 11–16 years in the lower class report more maternal control than middle-class girls [39]. However, to date, the father–son dyad, father–daughter dyad, mother–son dyad, and mother-daughter dyad have not received much attention.

The present study aimed to investigate whether fathers’ and mothers’ behavioral control is differentially related to the sociodemographic factors of adolescents and parents, as well as the gender differences in the association between parental factors and behavioral control. Based on the theoretical arguments and empirical findings presented above, this study raises eight hypotheses:

**Hypothesis** **1:** *There are significant adolescent gender differences in fathers’ and mothers’ behavioral control (Hypotheses 1a and 1b).*

**Hypothesis** **2:** *Adolescent age is significantly related to fathers’ and mothers’ behavioral control (Hypotheses 2a and 2b).*

**Hypothesis** **3:** *Adolescents’ number of siblings is significantly related to and fathers’ and mothers’ behavioral control (Hypotheses 3a and 3b).*

**Hypothesis** **4:** *There are paternal gender differences in parental behavioral control.*

**Hypothesis** **5:** *Fathers’ education level and income are significantly related to fathers’ behavioral control (Hypotheses 5a and 5b).*

**Hypothesis** **6:** *Mothers’ education level and income are significantly related to mothers’ behavioral control (Hypotheses 6a and 6b).*

**Hypothesis** **7:** *There are differences in the relationships between sociodemographic factors and fathers’ behavioral control by adolescent gender*.

**Hypothesis** **8:** *There are differences in the relationship between sociodemographic factors and mothers’ behavioral control by adolescent gender*.

## 2. Materials and Methods

### 2.1. Participants and Procedure

A sample of 916 adolescents aged 11–15 years old (mean = 12.98 ± 0.72 years) participated in a cross-sectional survey. This study included 487 boys (53.5%) and 429 girls (46.5%). The survey was conducted at two public junior high schools in two cities located in Shandong Province, China, in December 2023. All the students in grades 7–8 from the two schools were invited. After approval by the Human Ethics Committee of Taylor’s University, consent forms were obtained from the parents and students. Finally, the adolescents completed the questionnaires in their classes according to the researcher’s guidelines in approximately 20–30 min.

### 2.2. Measures

#### 2.2.1. Adolescent and Parent Factors

Adolescents reported their age, gender, number of siblings, father’s and mother’s age, education level, and monthly income. The possible educational categories based upon the Chinese educational system were as follows: from “primary school or below” to “postgraduate degree or above” were scored 1~6 (see Table 1). Higher scores indicated higher levels of parental education. Based on the Shandong Provincial Bureau of Statistics [40], the per capita monthly disposable income was CNY 2975/person. This study divided parents’ monthly income into 12 categories. Adolescents reported their parents’ monthly income from “below ¥1000” to “Above ¥100,000”, scoring from 1 to 12 (see Table 1). Higher scores indicated higher levels of parental income. The group’s information is presented in Table 1.

#### 2.2.2. Fathers’ and Mothers’ Behavioral Control

The Chinese version of the parental behavioral control questionnaire [3] was employed to evaluate fathers’ and mothers’ behavioral control. There were 16 items and two dimensions: parental solicitation and parental restriction (e.g., “My father/mother requires me to inform him about what I have done at school.”). Students responded to items on a 5-point Likert-scale from “never” to “always,” with higher mean scores showing higher levels of behavioral control. Confirmatory factor analysis indicated that the measurement model for fathers’ behavioral control had good psychometric properties (χ^2^/*df* = 3.261; RMSEA = 0.050; NFI = 0.94; CFI = 0.980), as well as mothers’ behavioral control (χ^2^/*df* = 2.943; RMSEA = 0.046; NFI = 0.991; CFI = 0.994). The scale obtained good Cronbach’s alpha values of 0.924 for fathers and 0.938 for mothers.

### 2.3. Data Analysis

First, descriptive analysis was conducted using SPSS 27.0 to compute the frequency, percentage, mean, and standard deviation of sociodemographic variables and the mean and standard deviation of parental behavioral control.

Next, Student’s *t*-tests were performed to compare gender differences in parental behavioral control and Cohen’s d was employed to evaluate effect size using SPSS 27.0. The dependent sample t-test was used to examine adolescent gender differences, and the paired sample t-test was used to examine parental differences in behavioral control.

Third, to examine the relationships between adolescent and parental factors and parental behavioral control, Pearson’s correlation analysis and multiple linear regression analysis were conducted using SPSS 27.0. With reference to the existing literature, the sex of an adolescent was converted into a dummy variable (boys = 1) [41]. The adolescent age, number of siblings, parental age, parental education level, and parental monthly income were viewed as continuous variables [41,42]. Before conducting correlation and regression analysis, the normality of the fathers’ and mothers’ parental behavioral control was checked. The scores of skewness (−0.107 and −0.477) and kurtosis (−0.288 and −0.067) showed that they were considered normally distributed, using the criteria of skewness ≤ 2 and kurtosis ≤ 7 [43]. Multiple linear regression was performed using the stepwise method. The variance inflation factors (VIFs) were acceptable, scoring from 1.00 to 1.14.

Subsequently, to examine the adolescent gender differences in the relationships between sociodemographic factors and parental behavioral control, two-group comparison analysis of the structural equation modeling (SEM) model was conducted using AMOS 28.0. SEM could indicate unobserved and latent factors in the whole relationship and account for the measurement errors in the analysis. Using SEM, both the measurement model and structural model were evaluated. Moreover, SEM could describe the entire set of relationships to define the research model [44]. The fit of the SEM model was evaluated using the following criteria: a significant value for χ^2^ indicated a poor fit between the model and the data; the comparative fit index (CFI) ≥ 0.95; the normed fit index (NFI) ≥ 0.95; and the root mean square error of approximation (RMSEA) < 0.08 [45,46]. To test for differences in the model between adolescent boys and girls, the unconstrained model allowed the structural paths to vary between boys and girls. Then, the measurement model was established with equal measurement weights across different gender groups. The constrained model constrained all parameter estimates for boys and girls to be equal. If the result of the Chi-square difference test (between the constrained model and measurement model) was significant, there were significant differences between the adolescent boys’ and girls’ models.

## 3. Results

### 3.1. Descriptive Findings and Correlations

Firstly, the descriptive characteristics of the sample are displayed in Table 1. The table indicates that the numbers of boys and girls were close. There were 487 adolescent boys (53.5%). A total of 107 (11.7%) adolescents had no siblings. As shown in Table 1, 663 adolescents (72.4%) had one sibling. Most adolescents had one sibling. A total of 125 adolescents (13.6%) had two siblings, while 21 adolescents (2.3%) had three or more siblings.

Fathers were aged 33–66 years (mean = 42.27 ± 5.65 years), and mothers were aged 32–59 years (mean = 41.47; SD = 5.82 years). Table 1 shows that the fathers’ education level and monthly income were slightly higher than those of the mothers. The proportion of the fathers’ monthly income was largest at CNY 5000–7000 (26.8%), and the proportion of the mothers’ monthly income was largest at CNY 3000–5000 (25.8%). Since most of the families in this study had two children, this result is close to the data from the Shandong Provincial Bureau of Statistics [40].

The dependent sample t-test was conducted to compare the adolescent gender differences in the fathers’ and mothers’ behavioral control. Table 2 reveals that there was a significant difference in the mean scores for boys’ and girls’ perception of their fathers’ behavioral control, with *t* (914) = 2.09; *p* < 0.05; and *d* = 0.84. These findings indicate that early adolescent boys significantly perceived greater behavioral control from their fathers than girls. Hypothesis 1a was supported. There were no significant differences between the boys’ and girls’ perceptions of their mothers’ parental behavioral control.

A paired-sample *t*-test was conducted to compare the differences between fathers’ and mothers’ behavioral control. Table 3 shows that the mean score of paternal behavioral control was significantly lower than that of maternal behavioral control, with *t* (915) = −21.97; Cohen’s *d* = 0.75; and *p* < 0.001. The findings suggest that the level of fathers’ behavioral control was significantly lower than that of mothers’ behavioral control.

Table 4 reports that the number of siblings was significantly and positively correlated with mothers’ behavioral control (*r* = 0.07; *p* < 0.05). The findings indicated that the more siblings there were, the higher the level of mothers’ behavioral control was likely to be. Mothers’ education level and monthly income were significantly and negatively correlated with mothers’ behavioral control (*r* = −0.08; *p* < 0.05; *r* = −0.07; *p* < 0.05). The findings indicated that the higher the levels of mothers’ education level and monthly income were, the lower the level of mothers’ behavioral control was likely to be.

### 3.2. Regression Analysis

This study conducted two multiple regression analyses by stepwise to determine the unique effects of adolescent and parental factors on fathers’ and mothers’ behavioral control. Adolescents’ gender, age, and number of siblings and parental age, education level, and monthly income were used as predictors, and parental behavioral control was used as the dependent variable.

Table 5 shows that only adolescent gender entered the significant model in the model for fathers (*R* = 0.07; *R*^2^ = 0.005; *F* = 4.96; *p* = 0.03), with *β* = 0.07; *SE* = 0.06; *t* = 2.23; and *p* < 0.05. It indicated that fathers’ behavioral control might vary by adolescent gender.

Table 6 indicates that only mothers’ education level entered the significant model in the model for mothers (*R* = 0.07; *R*^2^ = 0.005; *F* = 4.60; *p* = 0.03), with *β* = −0.07; SE = 0.06; *t* = −2.14; and *p* < 0.05. It indicated that mothers’ education level might be significantly related to mothers’ behavioral control. Hypothesis 6a was supported.

### 3.3. Structural Equation Model Analysis

Finally, to examine the adolescent gender differences in relationships, this study conducted multi-group analysis by SEM. Adolescent and parental factors were the observed and independent variables, and fathers’ and mothers’ behavioral control were the latent and dependent variables. Parental behavioral control was measured by parental solicitation and parental restriction. A two-group model was constructed with adolescents’ gender as the group variable.

**Figure 1:**
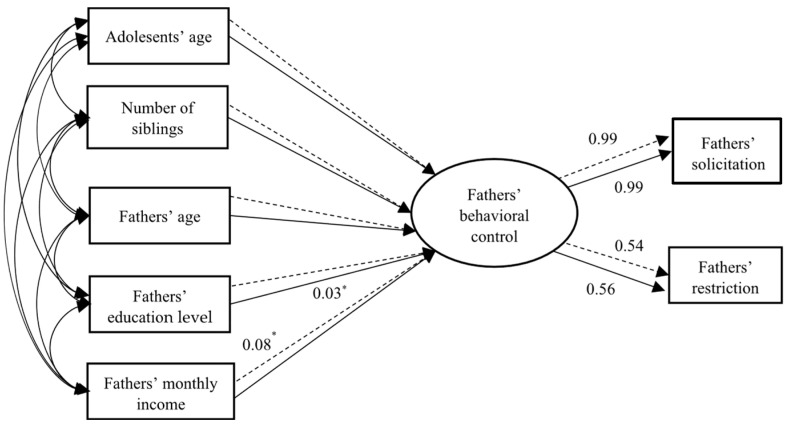
Adolescent gender differences in the relationships between sociodemographic factors and fathers’ behavioral control. Only significant path coefficients are shown in the model. Note: * *p* < 0.05; solid lines are for boys, and dashed lines are for girls.

The boys’ and girls’ models for fathers’ behavioral control are displayed in Figure 1. These two models’ fits were good: *χ*^2^/*df* = 1.467; *p* = 0.209; CFI = 0.994; NFI = 0.983; RASEA = 0.031; *χ*^2^/*df* = 1.568; *p* = 0.180; CFI = 0.990; NFI = 0.976; and RASEA = 0.036. To examine differences between the boys’ and girls’ models for fathers’ behavioral control, this study constructed an unconstrained model, F1. Next, the model F2 was constructed based on F1 with equal measurement weights across different gender groups. Both the F1 (*χ*^2^/*df* = 1.520; CFI = 0.992; NFI = 0.996; RASEA = 0.000) and F2 (*χ*^2^/*df* = 1.394; CFI = 0.993; NFI = 0.959; RASEA = 0.021) models had good fits. Based on F2, a constrained model, F3, was constructed with all parameter estimates for boys and girls being equal. Multi-group comparison analyses revealed significant differences between the F2 and F3 models [Δ*χ*^2^ (5) = 23.939; *p* < 0.01], indicating that there were adolescent gender differences in the relationships between the predictors and fathers’ parental behavioral control.

As shown in Figure 1 and Table 7, fathers’ education level had a significant and positive relationship with fathers’ behavioral control reported by boys (*β* = 0.03; *SE* = 0.03; *p* < 0.05). Fathers’ monthly income had a significant and positive relationship with fathers’ behavioral control reported by girls (*β* = 0.08; *SE* = 0.02; *p* < 0.05).

**Figure 2:**
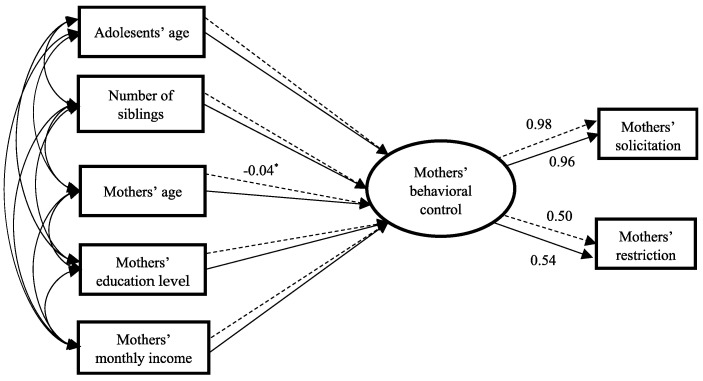
Adolescent gender differences in the relationships between sociodemographic factors and mothers’ behavioral control. Only significant path coefficients are shown in the model. Note: * *p* < 0.05; solid lines are for boys, and dashed lines are for girls.

The boys’ and girls’ models for mothers’ behavioral control are displayed in Figure 2. These two models’ fits were good: *χ*^2^/*df* = 0.449; *p* = 0.773; CFI = 0.999; NFI = 0.996; RASEA = 0.000; *χ*^2^/*df* = 2.066; *p* = 0.082; CFI = 0.987; NFI = 0.977; and RASEA = 0.050. To examine differences between the boys’ and girls’ models for mothers’ behavioral control, this study constructed an unconstrained model, M1. Next, the model M2 was constructed based on M1 with equal measurement weights across different gender groups. Both the M1 (*χ*^2^/*df* = 1.264; CFI = 0.997; NFI = 0.982; RASEA = 0.017) and M2 (*χ*^2^/*df* = 1.092; CFI = 0.999; NFI = 0.994; RASEA = 0.010) models had good fits. Based on M2, a constrained model, M3, was constructed with all parameter estimates for boys and girls being equal. Multi-group comparison analyses revealed significant differences between the M2 and M3 models [Δ*χ*^2^ (5) = 16.840; *p* < 0.01], indicating that there were adolescent differences in the relationships between the predictors and mothers’ parental behavioral control.

Figure 2 and Table 7 demonstrate that mothers’ age had a significant and negative relationship with mothers’ behavioral control reported by girls (*β* = −0.04; *SE* = 0.01; *p* < 0.05).

## 4. Discussion

This study aimed to determine which specific adolescent and parent sociodemographic factors might contribute to fathers’ and mothers’ behavioral control. The findings indicated that adolescent gender correlated with fathers’ behavioral control. Parental gender correlated with parental behavioral control. Mothers’ education level correlated with mothers’ behavioral control. Moreover, there were adolescent gender differences in the relationships between fathers’ education level and behavioral control, fathers’ monthly income and behavioral control, and mothers’ age and mothers’ behavioral control.

First, this study found that adolescent gender correlated with fathers’ behavioral control but not mothers’ behavioral control. The results indicated that adolescent boys perceived more behavioral control from their fathers than girls. These findings reinforced the cross-sectional findings that adolescent boys perceive a higher level of behavioral control from their fathers [18], which can be explained by traditional Chinese culture. A traditional Chinese saying goes “*zi bu jiao*, *fu zhi guo* (It’s the father’s fault that the son wasn’t taught well)”. In addition, there is a traditional Chinese practice in which fathers tend to have higher expectations of their sons than daughters [17,18]. Therefore, fathers are primarily involved in disciplining and restricting their sons, expecting them to achieve social success and honor their families [18]. However, there were no significant adolescent gender differences in mothers’ behavioral control. This does not confirm previous findings further, in which Chinese adolescent girls experience a higher level of maternal behavioral control than boys [16,17]. More future investigations may need to be conducted to clear these inconsistent findings. Moreover, the results of this study are in line with the reciprocal role theory [47]. This framework indicates that fathers prefer to treat boys and girls differently than mothers. For example, fathers value the independence of boys but expect girls to be more empathetic.

In terms of adolescents’ age, this study could not determine whether it was correlated with both fathers’ and mothers’ behavioral control. However, a consistent conclusion was reached among Chinese adolescents in Hong Kong [48], while the opposite was reported in a longitudinal study [17]. This study employed a cross-sectional design. Different designs may lead to different findings. Longitudinal studies are more pertinent for determining whether parental behavioral control varies with adolescent age. Future studies should take into account longitudinal designs.

The number of siblings did not significantly relate to fathers’ and mothers’ behavioral control, and it has received less attention due to China’s previous fertility policies. To the best of our knowledge, the present study may be the first to examine the correlation between the number of siblings and parental behavioral control in China. Based on the results of this study, fathers’ and mothers’ behavioral control may not have varied by adolescents’ number of siblings in the current sample.

Regarding parental factors, parental gender was one of the critical correlates of parental behavioral control. The present study reported that mothers might have performed more behavioral control than fathers. Both findings in the West [24] and the conclusion in China [17,18] reveal a similar idea. In a systematic review, fathers cared less about their children than mothers, and mothers showed more behavioral control than fathers [23]. Since fathers usually play a powerful and protective role in the family, they are inclined to apply parenting practices such as harsh punishments and authoritarianism. However, mothers usually take on a nurturing role and they more often need to employ authoritative parenting styles such as providing support and establishing norms [23]. A similar phenomenon has been observed in Chinese culture. Mothers are expected to nurture and monitor most life events of their children [18]. As such, Chinese mothers are more behaviorally controlling than Chinese fathers.

Overall, fathers’ age, education level, and monthly income did not correlate with their behavioral control in this study. However, after the group comparison analysis, this study found positive relationships between fathers’ education level and parental behavioral control reported by adolescent boys and fathers’ monthly income and parental behavioral control reported by adolescent girls. These findings echo those of the family stress model [35]. Fathers with higher financial statuses and education levels may be more likely to be involved in effective parenting. In general, fathers’ parenting practices may be powerful and reasonable [23], such as teaching social discipline and caring for their children’s extracurricular activities [49]. However, the adolescent gender differences were complex. The linkage between paternal education level and behavioral control in adolescent boys confirmed the father–son dyad. This provided some support for the previous findings. Interactions between same-gender parents and children may be prioritized [17]. As fathers become more educated, they realize the significance of positive parenting. In terms of the link between paternal monthly income and behavioral control, a father–daughter dyad was found. In other words, fathers at lower income levels might be prone to having less control over their daughters’ behavior. Fathers with low incomes facing financial pressure may struggle to care for their daughters. They may even have patriarchal attitudes toward their sons and may not concern themselves with their daughters. Studies have shown that sons are preferred in certain poor regions in China [50]. When socioeconomic status changes, this preference also changes. An increase in a father’s income increases the educational level of girls but has no effect on boys [51]. This echoes the findings of the present study.

Among the maternal factors, only education level may have had a negative relationship with mothers’ behavioral control. This is in line with the findings among urban African American mothers [27]. This may be due to the fact that mothers with higher levels of education tend to be more supportive, emotionally warm, and democratic without a high level of restrictions [31,49]. Yet, there is limited evidence supporting this finding. Further investigation is necessary to determine the direction of the effect of maternal education level on behavioral control.

Although the present study found that mothers’ age did not predict maternal behavioral control in the overall sample, there was a negative relationship between mothers’ age and behavioral control, as reported by adolescent girls but not boys. Older mothers were believed to be more responsive and sensitive [52]. They may have been more prone to providing emotional support and autonomy than exerting behavioral control. The association between mothers’ age and girls’ maternal behavioral control also illustrated the close interaction between mothers and daughters, once again confirming same-gender parent–child dyads. These findings provide further evidence for gender socialization theory [38].

Overall, the coefficients of these effects were low in this study. Although the roles of these demographic and sociological factors are relatively small, they should not be overlooked. This may be seen as good news for Chinese parents [42], as socio-demographic factors are difficult to change. If the effect sizes of these factors are large, the development and interventions of parenting behaviors are limited. On the other hand, the number of early adolescent cohorts is large in China. In 2023, there were 52,436,900 junior high school students in China [53]. Although the rate of explanation for the effect in this study was low, it is only significant for a very small proportion of the group, which is in the thousands in its number. The results of this study may be of value to this group of adolescents. Future research may specifically explore the selection of adolescents with a high socioeconomic status and low socioeconomic status with a view to developing more precise prevention and intervention programs.

From the findings of the present study, several other implications could be described. Theoretically, it may be insightful to separately identify the potential correlates of fathers’ and mothers’ behavioral control in China. The current investigation adds to the knowledge of parental behavioral control according to gender of adolescents and parents. Particularly, few studies have focused on the effects of the number of siblings on parental behavioral control, to the best of our knowledge. Parenting in multiple-child situations is becoming more common in China as fertility policies change. Although this study did not find a significant effect of the number of siblings, the initial attempts of this study provide a direction for future parenting studies.

Practically, the findings may help fathers and mothers to obtain advice on improving the effectiveness of their parenting. For instance, fathers may need to increase their parenting involvement and employ an appropriate degree of behavioral control. Additionally, when family counselors and social workers conduct parenting interventions, they need to be careful not to ignore the background information of the parents and children. Parental behavioral control development interventions may target less educated mothers, less educated fathers with sons, low-income fathers with daughters, and young mothers with daughters.

Inevitably, this study has some limitations, despite some advances. The first is its cross-sectional design, which is not capable of determining the direction of causality. Hence, future studies should conduct two or more waves of surveys in a longitudinal study to identify whether parental and child background information serves as an antecedent of parental behavioral control. Second, a single-source bias may appear when the data are only from adolescent reports. Parents’ perspectives may differ from those of adolescents and should be examined in further studies. Third, the diversity of the samples in this study was limited. Parenting may vary between different ethnic groups, family structures, residences, and so on [54]. Thus, further work is critical. Lastly, it is inevitable to note that socio-demographic factors explained much less of the variance. The findings of this study should be applied with great caution. Other crucial related factors, such as parental personality traits, parental beliefs and emotions, parent–child relationships, and so on, may be the primary targets in future studies.

## 5. Conclusions

Through a survey of Chinese adolescents, our findings indicated that adolescent gender was a correlate of fathers’ behavioral control, and parental gender was a crucial correlate of parental behavioral control. Fathers’ education level may have been a correlate of fathers’ behavioral control in adolescent boys. Fathers’ income may have been a correlate of fathers’ behavioral control in adolescent girls. Meanwhile, mothers’ education level may have been a correlate of mothers’ behavioral control, and mothers’ age may have been a correlate of mothers’ behavioral control in adolescent girls. These findings deepen the understanding of mothers’ and fathers’ parenting and offer practical implications for Chinese family interventions.

## Figures and Tables

**Table 1:** Information on adolescent and parental factors.

Factors	*n*	%		
Adolescent factor				
Gender				
Boy	487	53.5		
Girl	429	46.5		
Number of siblings				
None	107	11.7		
1	663	72.4		
2	125	13.6		
3 or more	21	2.3		
Parental factor	Father		Mother	
	*n*	%	*n*	%
Education level				
Primary school education and below	14	1.6	37	4.3
Secondary school education	298	34.5	347	40.2
High school education	165	19.1	151	17.5
Junior college education	221	25.6	166	19.2
Bachelor’s degree	149	17.3	146	16.9
Postgraduate degree or above	16	1.9	16	1.9
Monthly income (CNY)				
Below 1000	15	1.7	120	13.9
1000–2000	18	2.1	70	8.1
2000–3000	46	5.3	142	16.5
3000–5000	146	16.9	223	25.8
5000–7000	231	26.8	137	15.9
7000–10,000	191	22.1	94	10.9
10,000–15,000	97	11.2	34	3.9
15,000–20,000	51	5.9	12	1.4
20,000–30,000	31	3.6	14	1.6
30,000–50,000	17	2.0	5	0.6
50,000–100,000	11	1.3	3	0.3
Above 100,000	9	1.0	9	1.0

**Table 2:** Adolescent gender differences in parental behavioral control.

Variable	M ± SD		*t*	*d*
	Boys	Girls		
Fathers’ behavioral control	3.05 ± 0.84	2.94 ± 0.84	2.09 *	0.84
Mothers’ behavioral control	3.50 ± 0.89	3.59 ± 0.85	−1.46	0.87

Notes. M = mean; SD = standard deviation; *t* = Student’s t; *d* = Cohen’s d. * established level of significance: *p* < 0.05.

**Table 3:** Parental gender differences in behavioral control.

Variable	M ± SD		*t*	*d*
	Fathers	Mothers		
Behavioral control	3.03 ± 0.85	3.54 ± 0.89	−21.97 ***	0.75

Notes. M = mean; SD = standard deviation; *t* = Student’s t; *d* = Cohen’s d. *** established level of significance: *p* < 0.001.

**Table 4:** Correlations between adolescent factors, parental factors, and parental behavioral control by parental gender.

Variable	Behavioral Control (*r*)	
	Father	Mother
Adolescent factors		
Gender	0.07 *	−0.05
Age	0.04	0.05
Sibling status	0.03	0.07 *
Parental factors		
Age	−0.01	−0.01
Education level	0.05	−0.08 *
Monthly income	0.06	−0.07 *

Notes. * established level of significance: *p* < 0.05.

**Table 5:** Regression analysis of fathers’ behavioral control by adolescent and parental factors.

Variable				
Remove	Enter	*β*	*SE*	*t*
Constant		2.94	0.04	72.17 ***
*Adolescent factors*				
	Gender	0.07	0.06	2.23 *
Age		0.05		1.35
Sibling status		0.04		1.06
*Parental factors*				
Age		−0.02		−0.57
Education level		0.05		1.59
Monthly income		0.06		1.69
*R*	0.07			
*R* ^2^	0.005			
Adjusted *R*^2^	0.004			
*F*	4.96 *			

Notes. * established level of significance: *p* < 0.05; *** established level of significance: *p* < 0.001.

**Table 6:** Regression analysis of mothers’ behavioral control by adolescent and parental factors.

Variable				
Remove	Enter	*β*	*SE*	*t*
Constant		3.70	0.08	47.84 ***
Adolescent factors				
Gender		−0.05		−1.50
Age		0.06		1.73
Sibling status		0.06		1.76
Parental factors				
Age		−0.02		−0.46
	Education level	−0.07	0.02	−2.14 *
Monthly income		−0.04		−0.98
*R*	0.07			
*R* ^2^	0.005			
Adjusted *R*^2^	0.004			
*F*	4.60 *			

Notes. * established level of significance: *p* < 0.05; *** established level of significance: *p* < 0.001.

**Table 7:** Relationships between adolescent factors, parental factors, and parental behavioral control by adolescents’ gender.

	Boys		Girls			Boys		Girls	
Predictor	*β*	*SE*	*β*	*SE*	Predictor	*β*	*SE*	*β*	*SE*
Adolescent age	−0.00	0.04	−0.00	0.05	Adolescent age	0.07	0.03	0.02	0.05
Number of siblings	−0.02	0.06	−0.02	0.07	Number of siblings	0.05	0.03	−0.00	0.06
Fathers’ age	0.00	0.01	−0.05	0.01	Mothers’ age	−0.02	0.00	−0.04 *	0.01
Fathers’ education level	0.03 *	0.03	0.03	0.03	Mothers’ education level	0.01	0.01	−0.01	0.03
Fathers’ monthly income	0.00	0.02	0.08^*^	0.02	Mothers’ monthly income	−0.12	0.02	0.02	0.02

Notes. * established level of significance: *p* < 0.05.

## Data Availability

The data presented in this study are available on request from the corresponding author. The data are not publicly available due to ethical and privacy considerations to protect the confidentiality of the participants.
